# Transcriptome Analysis Describing New Immunity and Defense Genes in Peripheral Blood Mononuclear Cells of Rheumatoid Arthritis Patients

**DOI:** 10.1371/journal.pone.0006803

**Published:** 2009-08-27

**Authors:** Vitor Hugo Teixeira, Robert Olaso, Marie-Laure Martin-Magniette, Sandra Lasbleiz, Laurent Jacq, Catarina Resende Oliveira, Pascal Hilliquin, Ivo Gut, François Cornelis, Elisabeth Petit-Teixeira

**Affiliations:** 1 GenHotel-EA3886, Evry University - Paris 7 University Medical School, AutoCure European Consortium member, Evry, France; 2 Faculty of Medicine, University of Coimbra, Coimbra, Portugal; 3 CEA - IG – CNG, Functional genomic platform, Department of Translational Research, Evry, France; 4 UMR - AgroParisTech/INRA, MIA 518, Paris, France; 5 URGV- UMR - INRA 1165, CNRS 8114, Evry University, Evry, France; 6 Lariboisière Hospital, APHP, Paris, France; 7 Center for Neurosciences and Cell Biology, Faculty of Medicine, University of Coimbra, Coimbra, Portugal; 8 Sud Francilien Hospital Center, Corbeil-Essonnes, France; 9 CEA - IG - CNG - Department of Translational Research, Evry, France; 10 Clinical Genetics Unit, Lariboisière Hospital, APHP, Paris, France; Harvard Institute of Medicine, United States of America

## Abstract

**Background:**

Large-scale gene expression profiling of peripheral blood mononuclear cells from Rheumatoid Arthritis (RA) patients could provide a molecular description that reflects the contribution of diverse cellular responses associated with this disease. The aim of our study was to identify peripheral blood gene expression profiles for RA patients, using Illumina technology, to gain insights into RA molecular mechanisms.

**Methodology/Principal Findings:**

The Illumina Human-6v2 Expression BeadChips were used for a complete genome-wide transcript profiling of peripheral blood mononuclear cells (PBMCs) from 18 RA patients and 15 controls. Differential analysis *per* gene was performed with one-way analysis of variance (ANOVA) and *P* values were adjusted to control the False Discovery Rate (FDR<5%). Genes differentially expressed at significant level between patients and controls were analyzed using Gene Ontology (GO) in the PANTHER database to identify biological processes. A differentially expression of 339 Reference Sequence genes (238 down-regulated and 101 up-regulated) between the two groups was observed. We identified a remarkably elevated expression of a spectrum of genes involved in Immunity and Defense in PBMCs of RA patients compared to controls. This result is confirmed by GO analysis, suggesting that these genes could be activated systemically in RA. No significant down-regulated ontology groups were found. Microarray data were validated by real time PCR in a set of nine genes showing a high degree of correlation.

**Conclusions/Significance:**

Our study highlighted several new genes that could contribute in the identification of innovative clinical biomarkers for diagnostic procedures and therapeutic interventions.

## Introduction

Rheumatoid Arthritis (RA) is an autoimmune disease characterized by chronic and persistent joint synovial tissue inflammation associated with the destruction of affected joints [1]. The multifactorial nature of the disease provides a high RA heterogeneity with specific combinations of a genetic background and environmental factors that influence the susceptibility, severity and outcome of the disease [2]. The RA heterogeneity is demonstrated by the presence of distinct autoantibody specificities, like rheumatoid factor (RF) and anti-cyclic citrullinated peptide antibodies (ACPA) in the serum [3], [4], the differential responsiveness to treatment [5], [6], and the variability in clinical presentation. In addition, several gene expression profiling studies of synovial tissues and peripheral blood mononuclear cells (PBMCs) from RA patients showed marked variation in gene expression profiles that allowed to identify distinct molecular disease mechanisms involved in RA pathology [7], [8]. The relative contribution of the different mechanisms may vary among patients and in different stages of disease. Thus, the broad goals of expression profiling in RA are to (i) improve our understanding of the pathogenic mechanisms underlying RA, (ii) identify new drugs targets, (iii) assess activity of the disease, (iv) predict future outcomes, such as responsiveness therapy, overall disease severity, and organ specific risk and (v) develop new diagnostic tests [9].

PBMCs gene expression profiling allows both pathogenetic and pathophysiological processes identification as demonstrated in several types of diseases: cancer [10], asthma [11], systemic lupus erythematosus (SLE) [12], cardiovascular diseases [13] and psychiatric disorders [14]. Pathogenetic processes are primarily associated with the cause of a disease. Then, microarrays could lead to the identification of abnormal genes and gene activities that may not be only limited to PBMCs, but could occur in cells of pathological tissue as well. In contrast, pathophysiological changes in lymphocytic gene expression are considered an essentially normal reaction of the immune system to a pathological stimulus. Therefore, pathophysiological gene profiles may be shared in a variety of diseases, whereas pathogenetic gene expression is expected to be disease specific [15]. The differences in expression profiles provide opportunities to stratify RA patients based on molecular criteria that may require different treatment strategies.

Considering several comparative studies, Illumina and other microrray technologies have similar performances [16]–[18]. However, these studies showed that each approach was able to detect specific genes, meaning an increase in knowledge by each platform. To complete previous studies on RA with other microarrays [7], we applied Illumina large-scale gene expression profiling in PBMCs of RA patients to potentially gain insights into molecular mechanism of this disease. We identified new genes involved in different functional Immunity and Defense related mechanisms as pro-inflammation, anti-microbial activity, cellular stress and immunomodulatory functions in Rheumatoid Arthritis.

## Materials and Methods

### Study population

The study and all protocols presented here were approved by the Ethics Committees of Bicêtre and Saint Louis Hospitals (Paris, France) and all study participants provided written informed consent. All RA patients satisfied the revised criteria of the American College of Rheumatology [19] according to the rheumatologist in charge of the patient. A rheumatologist university fellow reviewed all clinical data. Characteristics of the 18 RA French Caucasian Patients are reported in **Table 1**. Among the control group consisted of 15 RA French Caucasian healthy individuals, 11 were females (mean ± Standard Deviation (SD) age at enrolment 56.9 ± 6.6). In all comparisons mentioned, the groups were age and sex-matched.

**Table 1:** Clinical and demographic characteristics of the RA patients.

Clinical features patients	RA Patients (*n* = 18)
Mean age (years)	60
Women (%)	72.3
Caucasian (%)	100
RF-positive (%)	88.9
ACPA-positive (%)	90.9 (out of 11 RA patients)
Mean disease duration (years)	8.6
Erosions (%)	63.2
Disease Activity Score 28 (DAS28) mean	5.22
Disease-Modifying Anti-Rheumatic Drugs use (%)	100
Anti-TNF therapy	0

### Isolation of total RNA

Peripheral blood (PB) was drawn in PAXgene RNA isolation tubes (PreAnalytix) from 18 patients and 15 controls. Total RNA was isolated from PBMCs using the PAXgene RNA isolation kit (PreAnalytix). Total RNA yield (ng) was determined spectrophotometrically using the NanoDrop ND-1000 (Wilmington). Total RNA profiles were recorded using a Bioanalyzer 2100 (Agilent). RNA integrity numbers were determined and the mean value was 8.07+/−0.51 SD and a Coefficient of Variation (CV) of 6.4%.

### Probe synthesis, hybridization and detection

cRNA was synthesized, amplified and purified using the Illumina TotalPrep RNA Amplification Kit (Ambion Inc.) following manufacturer recommendations. Briefly, 200 ng of RNA was reverse transcribed. After second strand synthesis, the cDNA was transcribed in vitro and cRNA labelled with biotin-16-UTP. Labelled probe hybridization to Illumina BeadChips human-6v2 was carried out using Illumina's BeadChip 6v2 protocol. These beadchips contain 48,701 unique 50-mer oligonucleotides in total, with hybridization to each probe assessed at ∼30 different beads on average. 22,403 probes (46%) are targeted at Reference Sequence (RefSeq) [20] transcripts and the remaining 26,298 probes (54%) are for other transcripts, generally less well characterized (including predicted transcripts).

**Figure 1:**
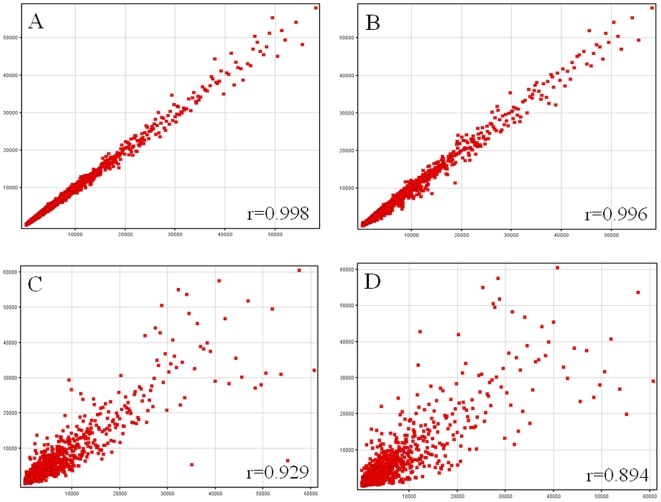
Scatter plots representation of signal intensities. Scatter plot of technical replicates: (A) same reference RNA undergoing two different hybridization or (B) same reference RNA from two different labeling runs. Typical scatter plots of data obtained from two patients (C) or a patient and a control (D). Pearson correlation coefficient is indicated in each scatter plot.

Beadchips were scanned on the Illumina BeadArray 500GX Reader using Illumina BeadScan image data acquisition software (version 2.3.0.13). Illumina BeadStudio software (version 1.5.0.34) was used for preliminary data analysis. To assess quality metrics of each run, several quality control procedures were implemented. Total RNA control samples were analyzed with each run. The Illumina BeadStudio software was used to view control summary reports, scatter plots of the total RNA control results from different days and scatter plots of daily run samples. The scatter plots compared control against control or sample against sample and calculated a correlation coefficient (**Figure 1**). Viewing the scatter plots determined whether controls across different days varied in quality, indicating a reduction in assay performance, and highlighted those samples that were of lower quality. The control summary report is generated by the BeadStudio software, which evaluates the performance of the built-in controls of the BeadChips across particular runs. This allows the user to look for variations in signal intensity, hybridization signal, background signal and the background to noise ratio for all samples analyzed in that run. Data are expressed as log2 ratios of fluorescence intensities of the experimental and the common reference sample. The Illumina data were then normalized using the ‘normalize quantiles’ function in the BeadStudio Software.

All microarray data reported in this study is described in accordance with MIAME guidelines and have been deposited in the National Center for Biotechnology Information Gene Expression Omnibus (GEO, http://www.ncbi.nlm.nih.gov/geo/) public repository, and they are accessible through GEO accession (GSE15573).

### Real-Time PCR

Total RNA was reverse transcribed using Superscript III and oligo(dT) primers (Invitrogen) according to the manufacturer's instructions. Real-time quantitative PCR was carried out using the SYBR-green master mix (Applied BioSystems) in an Mx 3005P thermocycler (Agilent). PCR conditions were 95°C for 10 min, followed by 40 cycles of 95°C for 15 sec and, 60°C for 1 min. At the end of the amplification reaction, melting curve analyses were performed to confirm the specificity as well as the integrity of the PCR products by the presence of a single peak. Gene-specific primers were designed inside or nearby the microarray sequence targeted, using Primer Express Software (PE Applied Biosystems). Primers sequences of all genes analyzed are provided in **Table S1**. Absence of cross contamination and primer dimer was checked on genomic DNA and water. From a list of 8 housekeeping genes (HKG), we chose *HMBS* and *ALDOA,* which meet the criteria of less variation between samples and compatible expression level with the studied genes. The geometric mean of HKG expression was used to normalize the expression of genes of interest [21]. Standard curves were generated from assays made with serial dilutions of reference cDNA to calculate PCR efficiencies (100%+/−15%, with r^2^ > = 0.997). Ct samples were transformed into quantity values using the formula (1+Efficiency)^Ct^. Only means of triplicate with a CV of less than 10% were analyzed. Inter-plate variation was below 8%.

### Statistical analysis

Statistical analysis on microarray data was performed using one-way analysis of variance (ANOVA) per gene where the normalized signals are explained by the patient status. One contrast was built to determine an expression difference between controls and RA patients [22]. Since the number of individuals is large, the residual variance was used to calculate the statistic test [23]. The raw *P* values were adjusted by the Benjamini-Hochberg procedure, which controls the False Discovery Rate (FDR) [24]. For the contrast, a gene is considered differentially expressed if the Benjamini-Hochberg-corrected *P* value is less than 0.05.

Genes that were expressed at significantly different levels between patients and controls were analyzed by supervised hierarchical clustering (uncentered correlation, complete linkage) [25] to visualize the correlation of co-expressed genes in Treeview (available at http://rana.lbl.gov/EisenSoftware.htm).

For an interpretation of the biological processes that are represented by the genes that show a significantly different level of expression in RA patients compared to the controls, we applied Gene Ontology analysis in the PANTHER database at http://www.pantherdb.org (Applied Biosystems) [26]. PANTHER uses the binomial statistics tool to compare our gene list to a reference list (NCBI: Homo sapiens genes) determining the statistically significant over- or under- representation of PANTHER biological process [27]. After, for each biological process in PANTHER, the genes associated with that term are evaluated according to the likelihood that their fold changes were drawn randomly from the overall distribution of fold changes. The Mann-Whitney U Test (Wilcoxon Rank-Sum Test) is used to determine the *P* value that, say, if specific biological process genes have random fold changes relative to the overall list of values that was input. A significant *P* value indicates that the distribution (fold change) for this category is non-random and different from the overall distribution [28]. In both statistical tests, processes with a *P* value <0.05 were considered significant after Bonferroni correction which was applied to adjust for multiple comparisons. Correlations between two set of data were measured using Pearson coefficient.

## Results

### Gene expression profiling in PMBCs of RA patients

**Figure 2:**
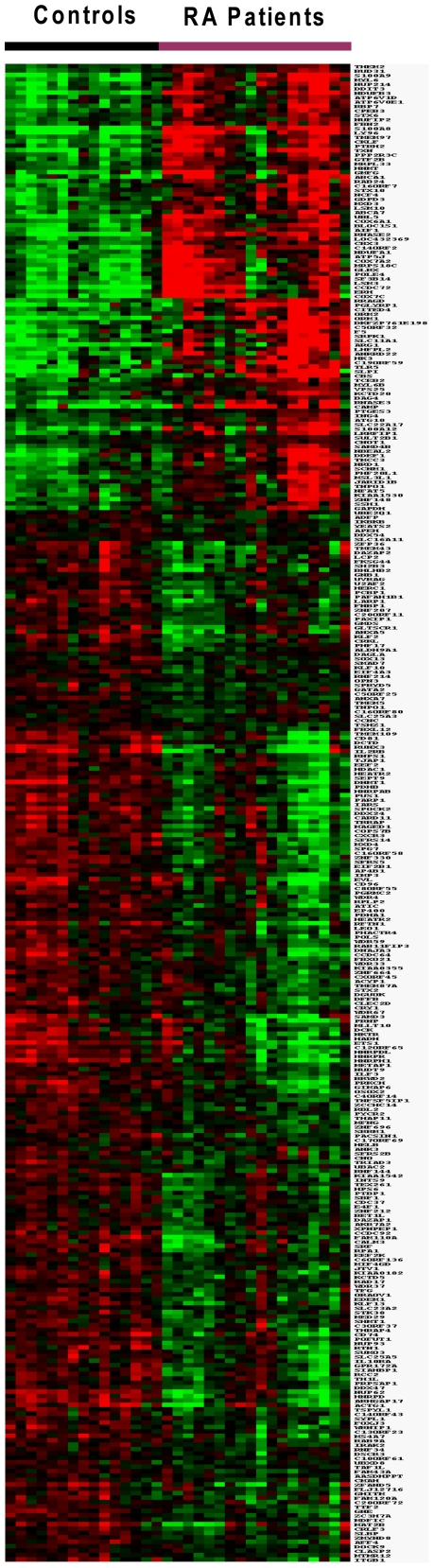
Cluster diagram of the expression of 339 significantly expressed genes in 18 RA patients and 15 controls. Genes are organized by hierarchical clustering based on overall similarity in expression patterns. Red represents relative expression greater than the median expression level across all samples, and green represents an expression level lower than the median. Black indicates intermediate expression.

Genome-wide transcriptional profiles of PBMCs from 18 RA patients and 15 age and sex-matched controls were measured on microarrays that contain 48,701 unique 50-mer oligonucleotides in total, with a mean ∼30 hybridizations *per* sequence. Data were analyzed using ANOVA. Using this test with a FDR of 5% we identified 380 transcripts with significant expression. The proportion of detected transcripts was substantially higher among RefSeq genes (91%) than non-RefSeq genes (9%), reflecting the greater degree of knowledge and certainty about the existence of RefSeq transcripts. Four genes, represented more than once in this list, were averaged from sequences with the same Unigene identifier. Significant difference in expression level between the two groups was observed for 339 RefSeq genes. Among them, 238 were downregulated (**Table S2**) and 101 were upregulated (**Table S3**). The significant gene expression differences between RA patients and controls were visualized in a cluster diagram (**Figure 2**).

### Genes upregulated in RA

**Figure 3:**
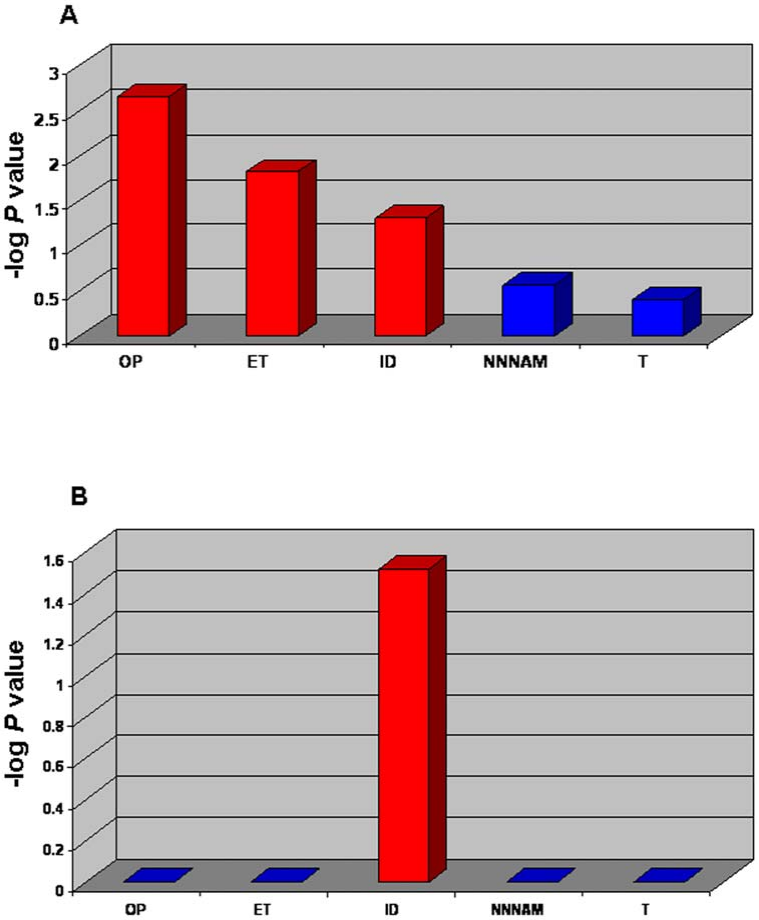
Gene ontology analysis of the most representative biological processes represented by the 101 up-regulated genes in RA patients. Gene Ontology (GO) analysis in the PANTHER database was applied for an interpretation of the biological processes that are represented by the genes showing a higher significantly different expression level in RA patients compared to the controls. A and B - Biological processes with a *P* value <0.05 (red bars) were considered significant after Bonferroni correction. The GO analysis *P*-value was plotted on the y axis versus biological processes on the x axis. A - The binomial statistics tool was used to compare our gene list to a reference list (NCBI: Homo sapiens genes) determining the statistically significant (*P* value) over- or under- representation of PANTHER biological process. B - The Mann-Whitney U Test was used to determine significant *P* values which indicate that the distribution (fold change) for each biological process is non-random and different from the overall distribution. OP - Oxidative Phosphorylation; ET - Electron Transport; ID - Immunity and Defense; NNNAM - Nucleoside, Nucleotide and Nucleic Acid Metabolism; T – Transport.

To categorize the up-regulated 101 genes into functional biological groups we used the PANTHER database (described in Methods section). We observed an elevated expression of a spectrum of genes involved in Immunity and Defense, nucleoside, nucleotide and nucleic acid metabolism, signal transduction, protein metabolism and modification, mRNA transcription, transport and developmental processes in the peripheral blood of RA patients compared to controls. Compared to a NCBI Homo sapiens reference list, the differentially up-regulated genes list revealed three biological processes significantly over represented (*P*<0.05) (**Figure 3A**). Then, the fold change of the genes associated to each biological process was compared to the overall distribution of fold changes. Immunity and Defense was the only significant functional biological process after Bonferroni correction (*P* = 0.03) (**Figure 3B**).

This cluster of genes involved in Immunity and Defense process contains the S100 family proteins S100 calcium-binding protein A8 (*S100A8*), *S1000A9* and *S100A12*, the orosomucoid family proteins *ORM1* and *ORM2*, as well as other inflammatory mediators like lymphocyte antigen 96 (*LY96*), cathelicidin antimicrobial peptide (*CAMP*), thioredoxin (*TXN*), allograft inflammatory factor 1 (*AIF1*), nuclear factor of activated T-cells 5 (*NFAT5*), *F5* (coagulation factor V), *SLC11A1* (solute carrier family 11, member 1) and *PGLYRP1* (peptidoglycan recognition protein 1). These genes are characterized in **Table 2**.

**Table 2:** Differentially up-regulated transcripts linked to Immunity and Defense biological process in RA patients.

Gene Bank	Name	Genome Location	GeneID (NCBI)	Transcript Identifier	Fold Change RA *vs* Controls
***S100A8***	S100 calcium binding protein A8	1q21	6279	NM_002964.3	2.9
***S100A9***	S100 calcium binding protein A9	1q21	6280	NM_002965.2	2.0
***S100A12***	S100 calcium binding protein A12	1q21	6283	NM_005621.1	1.8
***AIF1***	Allograft inflammatory factor 1	6p21.3	199	NM_001623.3	1.6
***TXN***	Thioredoxin	9q31	7295	NM_003329.1	2.1
***NFAT5***	Nuclear factor of activated T-cells 5, tonicity-responsive	16q22.1	10725	NM_173215.1	1.3
***CAMP (LL37)***	Cathelicidin antimicrobial peptide	3p21.3	820	NM_004345.3	2.5
***LY96 (MD-2)***	Lymphocyte antigen 96	8q21.11	23643	NM_015364.2	2.6
***ORM1***	Orosomucoid 1	9q31–q32	5004	NM_000607.1	2.7
***ORM2***	Orosomucoid 2	9q32	5005	NM_000608.2	1.5
***SLC11A1 (NRAMP1)***	Solute carrier family 11, member 1	2q35	6556	NM_000578.3	1.5
***PGLYRP1***	Peptidoglycan recognition protein 1	19q13.2–q13.3	8993	NM_005091.1	2.0
***F5***	Coagulation factor V	1q23	2153	NM_000130.4	1.7

### Genes downregulated in RA

Compared to the same NCBI Homo sapiens reference list, the mainstream of the genes that showed a lower expression in RA patients are linked to different biological processes such as nucleoside, nucleotide and nucleic acid metabolism, mRNA transcription and regulation, cell cycle, intracellular protein traffic. A small number of down-regulated genes were also involved in oncogenesis like runt-related transcription factor 3 (*RUNX3*), SMAD family member 7 (*SMAD7*), PHD finger protein 17 (*PHF17*) as well as interleukin-1 receptor-associated kinase 2 (*IRAK2*), interleukin 2 receptor, beta (*IL2RB*), *CD96* and SH2B adaptor protein 3 (*SH2B3*) related to immune functions. Therefore, in PANTHER database classification we found four significant functional biological processes in our 238 down-regulated genes list. However, no significant biological process was found (after Bonferroni correction) when we evaluate all the down-regulated genes associated to biological processes, according with their fold changes drawn randomly from the overall distribution of fold changes (data not shown).

### Real time PCR validation

**Figure 4:**
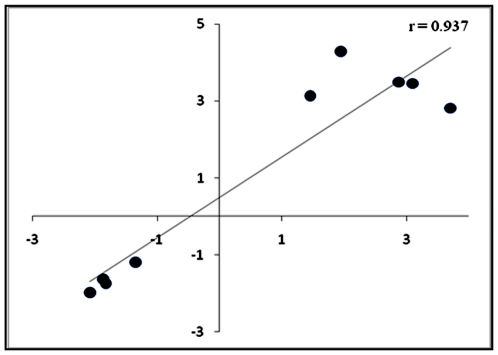
Correlation between microarray and real time PCR data. The scatter plot compares mean expression of RA patients/controls ratio for nine genes. Each point represents the RA patients/controls ratio from the microarray (y axis) and real time PCR (x axis). Pearson correlation coefficient is indicated in the scatter plot.

In all samples, we confirmed the expression of five up-regulated genes (*LY96*, *S100A12*, *ORM2*, *ORM1*, *RPL31)* and four down regulated genes (*IL2RB*, *DNMT1*, *RUNX3* and *IRF1*) in RA patients, by real-time PCR. From microarray and real time PCR data, we calculated the RA patients/controls ratio for each genes expression. The qPCR expression data of the nine genes showed a high correlation with the microarray expression data (r = 0.937) (**Figure 4**).

## Discussion

Microarray technology has been used to discriminate differences in gene expression profiles in tissues and PBMCs. Both synovial tissue and PBMCs have been used to evaluate differences in the gene expression profiles in RA [7], [8]. If expression-based profiling is to be of practical importance, sample accessibility becomes crucial. In this context, peripheral mononuclear cells are key sentinels of host defence, being used to identify novel disease mediators, disease variants and treatment responses [7], [29], [30].

**Figure 5:**
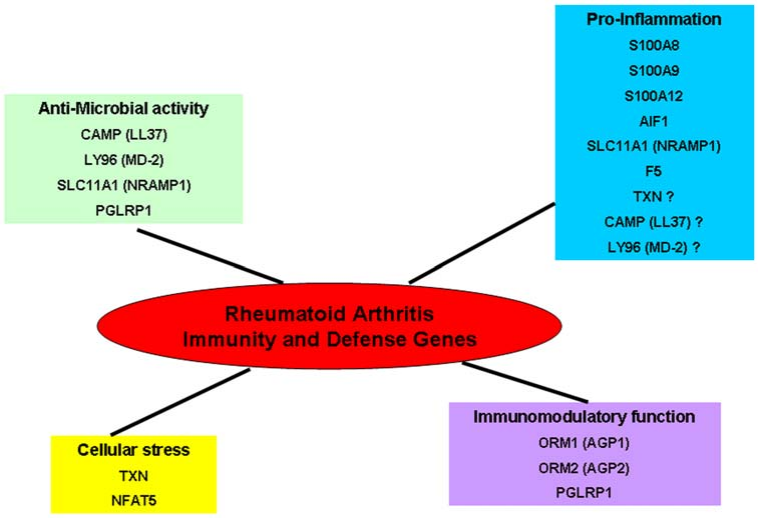
Biological functions of Immunity and Defense genes highlighted in our Rheumatoid Arthritis study. The genes differentially expressed in Immunity and Defense process are stratified in four functional related mechanisms: pro-inflammation, anti-microbial activity, oxidative and osmotic cellular stress and immunomodulatory functions.

Transcriptome studies using Illumina and other technologies showed that each approach was able to detect specific genes, meaning an increase in knowledge by each platform [16]–[18]. To complete previous studies on RA with Affymetrix or double colour microarrays, we decided to use Illumina technology. Our study did not confirm a specific expression for the genes regulated by interferon type I, as described in a RA large-scale expression profiling [8] (two colours analysis protocol using Stanford University microarrays) (data not shown). Our analysis revealed only one significantly increased biological mechanism: Immunity and Defense. This process was already highlighted by other studies in RA, as several genes that we described (*S100A8*, *S1000A9*, *S100A12* and *AIF1*). Additionally, our study identified new genes like *LY96*/*MD-2*, *NFAT5*, *TXN*, *CAMP*/*LL37*, *ORM1*, *ORM2*, *SLC11A1*, *PGLYRP1* and *F5*. These genes are involved in different functional Immunity and Defense related mechanisms as pro-inflammation, anti-microbial activity, oxidative and osmotic cellular stress and immunomodulatory functions (**Figure 5**).

The S100 calcium-binding proteins (S100A8, S100A9, and S100A12) are characterized by strong prevalence in cells of myeloid origin. Activated phagocytes expressing S100A8 and S100A9 proteins are among the first cells infiltrating inflammatory lesions in the synovium [31]. The expression of S100A8 and S100A9 was found to be strongest at the cartilage-pannus junction, which is the prime site of cartilage destruction and bone erosion in arthritis [32]. S100A12 is strongly expressed in inflamed synovial tissue, whereas it is nearly undetectable in synovia of control subjects or patients after successful treatment [33]. Further studies showed increased levels of S100A8/S100A9 and S100A12 concentrations in synovial fluid (SF) and serum in RA patients [34]–[36]. In addition, S100A9, S100A8 and S100A12 levels were associated with body mass index, presence of ACPA and RF and presence of ACPA, respectively [37]. Furthermore, several expression profiling studies in PBMCs showed a highly expression of *S100A8*, *S100A9* and *S100A12* in RA patients compared to controls [8], [38], [39]. Therefore, pro-inflammatory S100 proteins are attractive therapeutic targets for immune interventions in the treatment of RA.

Human allograft inflammatory factor-1 (AIF-1) is a Ca2+-binding EF-hand protein encoded within the major histocompatibility complex class (MHC) III region of chromosome 6. AIF-1 is produced by macrophages and lymphocytes, and its synthesis is mediated by several cytokines, such as interferon-γ (IFN-γ) [40]. Kimura et al. have demonstrated that AIF-1 is expressed in synovial and mononuclear cells in RA synovial tissue and increases proliferation of cultured synoviocytes and IL-6 production by these synoviocytes and PBMCs [41]. A recent study, showed an increased expression of *AIF1* mRNA in RA patients PMBCs compared with controls as well as in synovial macrophages in the lining layer of all the inflamed RA synovial membranes compared with non-inflamed OA controls [42]. AIF-1 plays therefore an important role in the pathogenesis of RA by affecting key processes such as the activation of synovial cell proliferation and the inflammatory cytokine cascade including IL-6 in joints and may represent a new molecular target in RA therapy.

The LY96 (MD-2) acts as an extracellular adaptor protein in the activation of TLR4 by the lipopolysaccharide (LPS) of Gram negative bacteria, a well-known inducer of the innate immune response [43]. Knockout studies in mice have demonstrated that MD-2 is indispensable for LPS responses [44]. Multiple evidences points to the potential importance of TLR signalling in RA pathogenesis by means of the presence of different TLR ligands and functional TLR receptors in inflamed joints from patients with RA [45]. *TLR4* mRNA is highly expressed in the synovium at early stages of RA as well as at later stages of the disease. *In vitro* stimulation of the RA synovial fibroblasts with TLR4 ligand (LPS) produces a wide range of proinflammatory cytokines, chemokines, and tissue destructive enzymes [46]. Recently, the potential value of TLR4 or signals derived from this receptor as therapeutic targets has become clearer - mainly as a result of studies in animal models of joint inflammation but also in human RA [47]. Interestingly, in our study, mRNA *TLR4* was up-regulated in RA patients compared to controls but not significantly. Thus, the TLR4-MD2-LPS complex could be involved in the activation of synovial fibroblasts and contribute to the development of synovial inflammation and joint destruction.

NFAT5, the primordial member of the NFAT family, is expressed by almost all cells and is activated in response to osmotic stress. In lymphocytes, NFAT5 controls the osmotic stress-induced expression of several cytokines, including tumour-necrosis factor (TNF) and lymphotoxin-β. *NFAT5*-deficient mice have impaired T-cell function under hyperosmotic conditions and decreased cellularity of the thymus and spleen [48]. In RA, *NFAT5* mRNA is expressed in proliferating RA-synovial fibroblasts (RA-SF) but not in nonproliferating RA-SF. Furthermore, *NFAT5* mRNA is expressed in RA synovium - but not in normal individuals - as well as at sites of bone destruction. NFAT5 could be then related not only with proliferation but also with the activation and invasion of RA-SF *in vivo* [49].

Oxidative stress to essential cell components caused by oxygen free radicals is generally considered as a serious mechanism in RA pathogenicity [50]. Thioredoxin (TXN), a cellular reducing catalyst induced by oxidative stress, is involved in the stimulation of the DNA-binding activity of NF-kb transcription factor [51]. Increased cytokine production driven by NF-κb can enhance expression of vascular adhesion molecules that attract leucocytes into the joint, as well as matrix metalloproteinases that help to degrade the extracellular matrix [50]. TXN concentrations were found significantly elevated in SF and serum of RA patients. The positive correlation between the SF TXN and the serum C Reactive Protein in the absence of a high concentration of SF TNF-α may indicate that TXN is involved in the prolongation and persistence of the RA inflammation, because high concentrations of TXN could stimulate NF-kB activation in the presence of the otherwise insufficient concentration of TNF-α [52]. Thus, TXN monitoring in RA patients could provide useful information regarding the extent of oxidative stress. Furthermore, truncated thioredoxin (Txn80) stimulates monocytes/macrophages to induce IL-12, implying that it is involved in immune inflammatory reactions directing Th1 immunity and IFN-γ production [53]. Moreover, Kim et al. have demonstrated that human TXN is a novel target gene induced by IFN-γ [54]. RA is a Th1-driven disease which has IFN-γ as characteristic Th1 cytokine and subsequently the *TXN* mRNA up-regulation in PBMCs could suggest an involvement in RA IFN-γ pathway.

CAMP (LL37) is an antimicrobial peptide which has a broad of antimicrobial activity. LL-37 has the potential to participate in the innate immune response both by killing bacteria and by recruiting a cellular immune response [55]. Gilliet and Lande recently found that LL37, overexpressed in psoriatic skin, is the key mediator of plasmacytoid dendritic cells (pDCs) activation in psoriasis. LL37 converts nonstimulatory self-DNA into a potent trigger of pDCs to produce IFN. pDCs respond to self-DNA if coupled with an antimicrobial peptide, suggesting that modified self-DNA drives autoimmunity in psoriasis by activating TLR9 [56]. In RA, recent studies have described pDCs as perpetuators of synovial inflammation and modulators of B cell responses in the synovial tissue [57]. The mRNA up-regulation of the *LL37* gene found in RA patients comparing to controls in our study could be due to an immunological response to infectious agents such as bacteria and viruses. Moreover the LL37 over-expression could be implicated in a pDCs-dependent mechanism involved in the perpetuation of RA inflammation through the abolition of self-tolerance and subsequent emergence of self-reactive lymphocytes.

Human alpha-1-acid glycoprotein (AGP) - also called orosomucoid - is a 37-kDa molecule consisting of a heavily glycosylated single polypeptide chain. Alpha-1-acid glycoprotein 1 (AGP1) and alpha-1-acid glycoprotein 2 (AGP2), coded by *ORM1* and *ORM2* genes respectively, are positive acute phase proteins [58], [59]. AGPs plasma concentration may increase several fold during acute phase reactions such as inflammation or chronic disease. AGPs have a strong immunomodulatory function [60]. It was shown that under pathological conditions not only the total concentration of AGPs but its glycosylation pattern may be altered [59]. Smith and colleagues have demonstrated that the AGPs populations in the serum and synovial fluid of RA patients are distinct in terms of glycosylation pattern. This discovery has direct functional significance since only the serum AGPs population is capable of blocking leucocyte adhesion [61]. Furthermore, Haston et al. have shown that AGPs can influence MMP-13 activity. It is hypothesized that AGPs may form part of a negative feedback mechanism which is inadequate to prevent disease progression in rheumatoid arthritis. These processes may exacerbate the increased turnover of collagen characteristic of the disease [62]. These results suggest an interesting role for AGP in RA pathogenesis.

*SLC11A1* (formerly called *NRAMP1*) is a gene that is important in macrophage-mediated natural resistance to a variety of intracellular pathogens. Exogenous and endogenous agents that mediate inflammation by activating the macrophage can cause NRAMP1 translocation to the membrane of the phagolysosome, where it serves as a cation transporter. The significant increase in iron deposition observed in the synovial membrane of rheumatoid arthritis patients, and foam cells in atherosclerotic lesions, could be attributable to NRAMP1 [63]. In human RA synovium NRAMP1 was detected in macrophages and neutrophils in the linning and subinitimal zone, as well as in inflammatory infiltrates, but was absent in fibroblasts [64]. NRAMP1 has also pleiotropic effects on macrophage function, including upregulation of chemokine/cytokine gene, TNFα, IL-1β, inducible nitric oxide synthase (iNOS), MHC expression as well as tumoricidal and antimicrobial activity. These effects are involved in resistance to infection and may also be involved in induction and maintenance of autoimmune disease [65]. TNFα and IL-1β play important roles in inflammation and tissue destruction of RA [66], [67]. Bacterial or viral infection may play a role in triggering the development of RA and TNFα and iNOS are key players in enhanced antimicrobial activity of activated macrophages [65].

PGRPs are innate immunity proteins, recognizing bacterial peptidoglycan, and acting in antibacterial immunity. PGRP-1 seems to belong to the innate immune arm of effectors molecules, such as antimicrobial peptides and C-type lectins, among others. This protein was shown to be almost exclusively present as a soluble protein in the granules of polymorphonuclear leucocytes (PMN) [68]. Saha et al. examined the immunomodulating activities of the PGRPs in a peptidoglycan-induced arthritis mice model. They showed that a systemic injection of peptidoglycan (PG) or muramyldipeptide (MDP) induces an acute arthritis of the joints of the feet in BalbC mice. Peptidoglycan-induced arthritis PGLYRP-1^−^/^−^ mice, had a MDP-induced activation of proinflammatory genes than WT mice. Moreover, PGLYRP-1^−^/^−^ mice have longer-lasting MDP-induced arthritis than WT mice. The anti-inflammatory function of PGLYRP-1 manifests itself only in the later stages of MDP-induced arthritis, which is consistent with the local release of PGLYRP-1 from PMN granules after PMNs' arrival into the mice foot [69]. These data point to that PGRP-1 could have a specialized but nevertheless significant role in signalling events like arthritis in mammals.

The multi-step coagulation complex system is activated by tissue factor (TF), which is exposed to blood. In this process, factor 5 (FV) is cleaved and activates factor Va (FVa). After several proteins interactions, the prothrombinase complex (FXa–FVa) converts prothrombin to thrombin which is generated in a large amount. Thrombin activates FV and FVIII and platelets converting fibrinogen to a fibrin clot [70]. Accumulation of fibrin in the RA synovium exceeds that in control tissue by a wide margin and represents one of the most striking pathologic features of rheumatoid synovitis. For some time, this fibrin deposition has been considered to be a serious contributor to permanent damage by maintaining a vicious circle of inflammation [71], [72]. In the extravascular coagulation at the arthritic synovial joint sequential activity of factor Xa (in the presence of cofactor Va) and of thrombin leads to fibrin deposition in the joint [73]. Thus, mRNA *F5* up-regulation in RA patients could enhance the production of factor V that under cleavage produces factor Va. This Va increase could lead to the augmentation of thrombin and consequently fibrin in RA joints.

In conclusion, our study highlighted several new genes (*LY96*/*MD*-*2, NFAT5*, *TXN, CAMP*/*LL37, ORM1*, *ORM2*, *SLC11A1*, *PGLYRP1* and *F5)* in PBMCs of RA patients that could contribute in the identification of innovative clinical biomarkers for diagnostic procedures and therapeutic interventions. Nevertheless, comparative analysis with another disease involving an inflammatory process could clarify the relation between the expression profiling and the pathophysiological processes specifically involved in Rheumatoid Arthritis.z

## Supporting Information

Table S1 Primers sequences of the nine genes analyzed by real-time PCR
(0.04 MB DOC)

Table S2 List of 238 downregulated genes differentially expressed between RA patients and controls
(0.28 MB DOC)

Table S3 List of 101 upregulated genes differentially expressed between RA patients and controls
(0.14 MB DOC)
