# Supplementary material for: Transcriptome Analysis Describing New Immunity and Defense Genes in Peripheral Blood Mononuclear Cells of Rheumatoid Arthritis Patients
Source: PLoS One. 2009 Aug 27;4(8):e6803. doi: 10.1371/journal.pone.0006803 (PMC2729373; doi:10.1371/journal.pone.0006803)
Supplement: Table S1 — Primers sequences of the nine genes analyzed by real-time PCR (0.04 MB DOC) [file pone.0006803.s001.doc]

**Table S1** Primers sequences of the nine genes analyzed by real-time PCR.

| **Gene symbol** | **Accession number** | **Forward primer** | **Reverse primer** |
| --- | --- | --- | --- |
| *DNMT1* | NM_001379 | AGCCCGAGAGAGTGCCTCA | GGCAGAACTAGTCCTTAGCAGCTT |
| *IL2RB* | NM_000878 | TTCATCATCTTAGTGTACTTGCTGATCA | TCTGGACGTCTCCTCCATGC |
| *IRF1* | NM_002198 | GGGTACCTACTCAATGAACCTGGA | TGTGAAGACACGCTGTAGACTCAG |
| *LY96* | NM_015364 | CGAGGATCTGATGACGATTACTCTT | ATTGTTGTATTCACAGTCTCTCCCTTC |
| *ORM1* | NM_000607 | CACCACCTACCTGAATGTCCAG | AGTTCTTCTCATCGTTCACGTCAA |
| *ORM2* | NM_000608 | AGAATGGGACCGTCTCCAGAT | TCTTCTCATCGTCCAGGTAGGAAC |
| *RPL31* | NM_000993 | CTTTCCTTCTCCCACAATCCTTC | TTCTTCTCGCCACCCTTCTTT |
| *RUNX3* | NM_004350 | AGGCTCACTCAGCACCACAAG | GAATGGGTTCAGTTCCGAGGT |
| *S100A12* | NM_005621 | AAAGGAGCTTGCAAACACCATC | CAGGCCTTGGAATATTTCATCAA |
| *ALDOA* | NM_000034 | CTGTCACTGGGATCACCTTCCT | AGGTTGATGGACGCCTCCT |
| *HMBS* | NM_000190 | ACCAAGGAGCTTGAACATGC | GAAAGACAACAGCATCATGAG |
