# Supplementary material for: Transcriptome Analysis Describing New Immunity and Defense Genes in Peripheral Blood Mononuclear Cells of Rheumatoid Arthritis Patients
Source: PLoS One. 2009 Aug 27;4(8):e6803. doi: 10.1371/journal.pone.0006803 (PMC2729373; doi:10.1371/journal.pone.0006803)
Supplement: Table S2 — List of 238 downregulated genes differentially expressed between RA patients and controls (0.28 MB DOC) [file pone.0006803.s002.doc]

**Table S2** List of 238 downregulated genes differentially expressed between RA patients and controls.

| **Gene Symbol** | **Definition** | **Transcript Identifier** |
| --- | --- | --- |
| *AASDHPPT* | Homo sapiens aminoadipate-semialdehyde dehydrogenase-phosphopantetheinyl transferase (AASDHPPT), mRNA. | NM_015423.2 |
| *ACTG1* | Homo sapiens actin, gamma 1 (ACTG1), mRNA. | NM_001614.2 |
| *ACYP1* | Homo sapiens acylphosphatase 1, erythrocyte (common) type (ACYP1), transcript variant 1, mRNA. | NM_001107.3 |
| *ADFP* | Homo sapiens adipose differentiation-related protein (ADFP), mRNA. | NM_001122.2 |
| *AFF4* | Homo sapiens AF4/FMR2 family, member 4 (AFF4), mRNA. | NM_014423.3 |
| *AKR7A2* | Homo sapiens aldo-keto reductase family 7, member A2 (aflatoxin aldehyde reductase) (AKR7A2), mRNA. | NM_003689.2 |
| *ALDH9A1* | Homo sapiens aldehyde dehydrogenase 9 family, member A1 (ALDH9A1), mRNA. | NM_000696.3 |
| *ANK3* | Homo sapiens ankyrin 3, node of Ranvier (ankyrin G) (ANK3), transcript variant 1, mRNA. | NM_020987.2 |
| *ANXA5* | Homo sapiens annexin A5 (ANXA5), mRNA. | NM_001154.2 |
| *ANXA7* | Homo sapiens annexin A7 (ANXA7), transcript variant 2, mRNA. | NM_004034.1 |
| *AP4B1* | Homo sapiens adaptor-related protein complex 4, beta 1 subunit (AP4B1), mRNA. | NM_006594.1 |
| *APEH* | Homo sapiens N-acylaminoacyl-peptide hydrolase (APEH), mRNA. | NM_001640.3 |
| *ARHGAP17* | Homo sapiens Rho GTPase activating protein 17 (ARHGAP17), transcript variant 2, mRNA. | NM_018054.4 |
| *ATIC* | Homo sapiens 5-aminoimidazole-4-carboxamide ribonucleotide formyltransferase/IMP cyclohydrolase (ATIC), mRNA. | NM_004044.4 |
| *BET1L* | Homo sapiens blocked early in transport 1 homolog (S. cerevisiae)-like (BET1L), transcript variant 2, mRNA. | NM_016526.4 |
| *BHLHB2* | Homo sapiens basic helix-loop-helix domain containing, class B, 2 (BHLHB2), mRNA. | NM_003670.1 |
| *BRWD2* | Homo sapiens bromodomain and WD repeat domain containing 2 (BRWD2), mRNA. | NM_018117.10 |
| *C10orf61* | Homo sapiens chromosome 10 open reading frame 61 (C10orf61), transcript variant 1, mRNA. | NM_001013840.1 |
| *C12orf65* | Homo sapiens chromosome 12 open reading frame 65 (C12orf65), mRNA. | NM_152269.2 |
| *C13orf23* | Homo sapiens chromosome 13 open reading frame 23 (C13orf23), transcript variant 1, mRNA. | NM_025138.3 |
| *C14orf43* | Homo sapiens chromosome 14 open reading frame 43 (C14orf43), transcript variant 1, mRNA. | NM_194278.3 |
| *C16orf58* | Homo sapiens chromosome 16 open reading frame 58 (C16orf58), mRNA. | NM_022744.1 |
| *C16orf80* | Homo sapiens chromosome 16 open reading frame 80 (C16orf80), mRNA. | NM_013242.2 |
| *C17orf69* | Homo sapiens chromosome 17 open reading frame 69 (C17orf69), mRNA. | NM_152466.1 |
| *C20orf11* | Homo sapiens chromosome 20 open reading frame 11 (C20orf11), mRNA. | NM_017896.2 |
| *C20orf72* | Homo sapiens chromosome 20 open reading frame 72 (C20orf72), mRNA. | NM_052865.2 |
| *C3orf37* | Homo sapiens chromosome 3 open reading frame 37 (C3orf37), transcript variant 2, mRNA. | NM_020187.2 |
| *C4orf14* | Homo sapiens chromosome 4 open reading frame 14 (C4orf14), mRNA. | NM_032313.2 |
| *C5orf25* | Homo sapiens chromosome 5 open reading frame 25 (C5orf25), mRNA. | NM_198567.2 |
| *C6orf136* | Homo sapiens chromosome 6 open reading frame 136 (C6orf136), mRNA. | NM_145029.1 |
| *C8orf55* | Homo sapiens chromosome 8 open reading frame 55 (C8orf55), mRNA. | NM_016647.2 |
| *CALM3* | Homo sapiens calmodulin 3 (phosphorylase kinase, delta) (CALM3), mRNA. | NM_005184.2 |
| *CARD11* | Homo sapiens caspase recruitment domain family, member 11 (CARD11), mRNA. | NM_032415.2 |
| *CCDC64* | Homo sapiens coiled-coil domain containing 64 (CCDC64), mRNA. | NM_207311.2 |
| *CCDC92* | Homo sapiens coiled-coil domain containing 92 (CCDC92), mRNA. | NM_025140.1 |
| *CCNC* | Homo sapiens cyclin C (CCNC), transcript variant 1, mRNA. | NM_005190.3 |
| *CD74* | Homo sapiens CD74 molecule, major histocompatibility complex, class II invariant chain (CD74), transcript variant 1, mRNA. | NM_001025159.1 |
| *CD81* | Homo sapiens CD81 molecule (CD81), mRNA. | NM_004356.3 |
| *CD96* | Homo sapiens CD96 molecule (CD96), transcript variant 1, mRNA. | NM_198196.2 |
| *CDC37* | Homo sapiens cell division cycle 37 homolog (S. cerevisiae) (CDC37), mRNA. | NM_007065.3 |
| *CLASP2* | Homo sapiens cytoplasmic linker associated protein 2 (CLASP2), mRNA. | NM_015097.1 |
| *CLEC2D* | Homo sapiens C-type lectin domain family 2, member D (CLEC2D), transcript variant 1, mRNA. | NM_013269.3 |
| *CMAH* | Homo sapiens cytidine monophosphate-N-acetylneuraminic acid hydroxylase (CMP-N-acetylneuraminate monooxygenase) (CMAH) on chromosome 6. | NR_002174.2 |
| *CNO* | Homo sapiens cappuccino homolog (mouse) (CNO), mRNA. | NM_018366.2 |
| *COPS7B* | Homo sapiens COP9 constitutive photomorphogenic homolog subunit 7B (Arabidopsis) (COPS7B), mRNA. | NM_022730.1 |
| *CRKL* | Homo sapiens v-crk sarcoma virus CT10 oncogene homolog (avian)-like (CRKL), mRNA. | NM_005207.2 |
| *CRLF3* | Homo sapiens cytokine receptor-like factor 3 (CRLF3), mRNA. | NM_015986.2 |
| *CRY1* | Homo sapiens cryptochrome 1 (photolyase-like) (CRY1), mRNA. | NM_004075.2 |
| *CXCR3* | Homo sapiens chemokine (C-X-C motif) receptor 3 (CXCR3), mRNA. | NM_001504.1 |
| *CXorf45* | Homo sapiens chromosome X open reading frame 45 (CXorf45), transcript variant 1, mRNA. | NM_001039210.1 |
| *DAGLA* | Homo sapiens diacylglycerol lipase, alpha (DAGLA), mRNA. | NM_006133.1 |
| *DAZAP1* | Homo sapiens DAZ associated protein 1 (DAZAP1), transcript variant 1, mRNA. | NM_170711.1 |
| *DAZAP2* | Homo sapiens DAZ associated protein 2 (DAZAP2), mRNA. | NM_014764.2 |
| *DCK* | Homo sapiens deoxycytidine kinase (DCK), mRNA. | NM_000788.1 |
| *DCTD* | Homo sapiens dCMP deaminase (DCTD), transcript variant 2, mRNA. | NM_001921.2 |
| *DDX24* | Homo sapiens DEAD (Asp-Glu-Ala-Asp) box polypeptide 24 (DDX24), mRNA. | NM_020414.3 |
| *DDX47* | Homo sapiens DEAD (Asp-Glu-Ala-Asp) box polypeptide 47 (DDX47), transcript variant 1, mRNA. | NM_016355.3 |
| *DDX54* | Homo sapiens DEAD (Asp-Glu-Ala-Asp) box polypeptide 54 (DDX54), mRNA. | NM_024072.3 |
| *DFFB* | Homo sapiens DNA fragmentation factor, 40kDa, beta polypeptide (caspase-activated DNase) (DFFB), mRNA. | NM_004402.2 |
| *DGUOK* | Homo sapiens deoxyguanosine kinase (DGUOK), nuclear gene encoding mitochondrial protein, transcript variant 2, mRNA. | NM_080918.1 |
| *DNAJA3* | Homo sapiens DnaJ (Hsp40) homolog, subfamily A, member 3 (DNAJA3), mRNA. | NM_005147.3 |
| *DNMT1* | Homo sapiens DNA (cytosine-5-)-methyltransferase 1 (DNMT1), mRNA. | NM_001379.1 |
| *DOCK9* | Homo sapiens dedicator of cytokinesis 9 (DOCK9), mRNA. | NM_015296.1 |
| *DSCR3* | Homo sapiens Down syndrome critical region gene 3 (DSCR3), mRNA. | NM_006052.1 |
| *E4F1* | Homo sapiens E4F transcription factor 1 (E4F1), mRNA. | NM_004424.3 |
| *EDEM1* | Homo sapiens ER degradation enhancer, mannosidase alpha-like 1 (EDEM1), mRNA. | NM_014674.1 |
| *EEF2* | Homo sapiens eukaryotic translation elongation factor 2 (EEF2), mRNA. | NM_001961.3 |
| *EEF2K* | Homo sapiens eukaryotic elongation factor-2 kinase (EEF2K), mRNA. | NM_013302.3 |
| *EIF2B1* | Homo sapiens eukaryotic translation initiation factor 2B, subunit 1 alpha, 26kDa (EIF2B1), mRNA. | NM_001414.2 |
| *EIF4A3* | Homo sapiens eukaryotic translation initiation factor 4A, isoform 3 (EIF4A3), mRNA. | NM_014740.2 |
| *EP400* | Homo sapiens E1A binding protein p400 (EP400), mRNA. | NM_015409.3 |
| *ETS1* | Homo sapiens v-ets erythroblastosis virus E26 oncogene homolog 1 (avian) (ETS1), mRNA. | NM_005238.2 |
| *EVL* | Homo sapiens Enah/Vasp-like (EVL), mRNA. | NM_016337.2 |
| *FAM110A* | Homo sapiens family with sequence similarity 110, member A (FAM110A), transcript variant 2, mRNA. | NM_207121.3 |
| *FAM120A* | Homo sapiens family with sequence similarity 120A (FAM120A), mRNA. | NM_014612.3 |
| *FAM43A* | Homo sapiens family with sequence similarity 43, member A (FAM43A), mRNA. | NM_153690.4 |
| *FBXL12* | Homo sapiens F-box and leucine-rich repeat protein 12 (FBXL12), mRNA. | NM_017703.1 |
| *FBXO21* | Homo sapiens F-box protein 21 (FBXO21), transcript variant 2, mRNA. | NM_015002.2 |
| *FKSG44* | Homo sapiens FKSG44 gene (FKSG44), mRNA. | NM_031904.3 |
| *FLJ12716* | Homo sapiens FLJ12716 protein (FLJ12716), transcript variant 1, mRNA. | NM_021942.4 |
| *FNBP1* | Homo sapiens formin binding protein 1 (FNBP1), mRNA. | NM_015033.2 |
| *FOXJ3* | Homo sapiens forkhead box J3 (FOXJ3), mRNA. | NM_014947.3 |
| *GATA2* | Homo sapiens GATA binding protein 2 (GATA2), mRNA. | NM_032638.3 |
| *GHITM* | Homo sapiens growth hormone inducible transmembrane protein (GHITM), mRNA. | NM_014394.2 |
| *GIMAP6* | Homo sapiens GTPase, IMAP family member 6 (GIMAP6), transcript variant 3, mRNA. | NM_001007224.1 |
| *GLTSCR1* | Homo sapiens glioma tumor suppressor candidate region gene 1 (GLTSCR1), mRNA. | NM_015711.2 |
| *GMDS* | Homo sapiens GDP-mannose 4,6-dehydratase (GMDS), mRNA. | NM_001500.2 |
| *GNB1* | Homo sapiens guanine nucleotide binding protein (G protein), beta polypeptide 1 (GNB1), mRNA. | NM_002074.2 |
| *GNE* | Homo sapiens glucosamine (UDP-N-acetyl)-2-epimerase/N-acetylmannosamine kinase (GNE), mRNA. | NM_005476.3 |
| *GPR172A* | Homo sapiens G protein-coupled receptor 172A (GPR172A), mRNA. | NM_024531.3 |
| *HADH* | Homo sapiens hydroxyacyl-Coenzyme A dehydrogenase (HADH), nuclear gene encoding mitochondrial protein, mRNA. | NM_005327.2 |
| *HDAC1* | Homo sapiens histone deacetylase 1 (HDAC1), mRNA. | NM_004964.2 |
| *HEATR2* | Homo sapiens HEAT repeat containing 2 (HEATR2), mRNA. XM_935824 XM_935825 | NM_017802.2 |
| *HELB* | Homo sapiens helicase (DNA) B (HELB), mRNA. | NM_033647.2 |
| *HERC1* | Homo sapiens hect (homologous to the E6-AP (UBE3A) carboxyl terminus) domain and RCC1 (CHC1)-like domain (RLD) 1 (HERC1), mRNA. | NM_003922.3 |
| *HNRPAB* | Homo sapiens heterogeneous nuclear ribonucleoprotein A/B (HNRPAB), transcript variant 2, mRNA. | NM_004499.3 |
| *HNRPD* | Homo sapiens heterogeneous nuclear ribonucleoprotein D (AU-rich element RNA binding protein 1, 37kDa) (HNRPD), transcript variant 3, mRNA. | NM_002138.3 |
| *HNRPDL* | Homo sapiens heterogeneous nuclear ribonucleoprotein D-like (HNRPDL), transcript variant 3, transcribed RNA. | NR_003249.1 |
| *HNRPH1* | Homo sapiens heterogeneous nuclear ribonucleoprotein H1 (H) (HNRPH1), mRNA. | NM_005520.1 |
| *HNRPR* | Homo sapiens heterogeneous nuclear ribonucleoprotein R (HNRPR), mRNA. | NM_005826.2 |
| *HPS6* | Homo sapiens Hermansky-Pudlak syndrome 6 (HPS6), mRNA. | NM_024747.4 |
| *IARS* | Homo sapiens isoleucyl-tRNA synthetase (IARS), transcript variant short, mRNA. | NM_002161.3 |
| *IKBKB* | Homo sapiens inhibitor of kappa light polypeptide gene enhancer in B-cells, kinase beta (IKBKB), mRNA. | NM_001556.1 |
| *IL10RA* | Homo sapiens interleukin 10 receptor, alpha (IL10RA), mRNA. | NM_001558.2 |
| *IL2RB* | Homo sapiens interleukin 2 receptor, beta (IL2RB), mRNA. | NM_000878.2 |
| *ILF3* | Homo sapiens interleukin enhancer binding factor 3, 90kDa (ILF3), transcript variant 2, mRNA. | NM_004516.2 |
| *IMP3* | Homo sapiens IMP3, U3 small nucleolar ribonucleoprotein, homolog (yeast) (IMP3), mRNA. | NM_018285.2 |
| *INTS9* | Homo sapiens integrator complex subunit 9 (INTS9), mRNA. | NM_018250.1 |
| *IRAK2* | Homo sapiens interleukin-1 receptor-associated kinase 2 (IRAK2), mRNA. | NM_001570.3 |
| *ITGB1* | Homo sapiens integrin, beta 1 (fibronectin receptor, beta polypeptide, antigen CD29 includes MDF2, MSK12) (ITGB1), transcript variant 1D, mRNA. | NM_033668.1 |
| *JTV1* | Homo sapiens JTV1 gene (JTV1), mRNA. | NM_006303.3 |
| *KCTD5* | Homo sapiens potassium channel tetramerisation domain containing 5 (KCTD5), mRNA. | NM_018992.2 |
| *KIAA0182* | Homo sapiens KIAA0182 (KIAA0182), mRNA. | NM_014615.1 |
| *KIAA0355* | Homo sapiens KIAA0355 (KIAA0355), mRNA. | NM_014686.3 |
| *KIAA1542* | Homo sapiens CTD-binding SR-like protein rA9 (KIAA1542), mRNA. | NM_020901.1 |
| *KLF10* | Homo sapiens Kruppel-like factor 10 (KLF10), transcript variant 1, mRNA. | NM_005655.1 |
| *KLF13* | Homo sapiens Kruppel-like factor 13 (KLF13), mRNA. | NM_015995.2 |
| *KLF2* | Homo sapiens Kruppel-like factor 2 (lung) (KLF2), mRNA. | NM_016270.2 |
| *LARP1* | Homo sapiens La ribonucleoprotein domain family, member 1 (LARP1), transcript variant 2, mRNA. | NM_033551.2 |
| *LCP2* | Homo sapiens lymphocyte cytosolic protein 2 (SH2 domain containing leukocyte protein of 76kDa) (LCP2), mRNA. | NM_005565.3 |
| *LEO1* | Homo sapiens Leo1, Paf1/RNA polymerase II complex component, homolog (S. cerevisiae) (LEO1), mRNA. | NM_138792.2 |
| *MAGED1* | Homo sapiens melanoma antigen family D, 1 (MAGED1), transcript variant 3, mRNA. | NM_001005332.1 |
| *MAT2B* | Homo sapiens methionine adenosyltransferase II, beta (MAT2B), transcript variant 1, mRNA. | NM_013283.3 |
| *MDFIC* | Homo sapiens MyoD family inhibitor domain containing (MDFIC), mRNA. | NM_199072.3 |
| *MED29* | Homo sapiens mediator complex subunit 29 (MED29), mRNA. | NM_017592.1 |
| *METAP1* | Homo sapiens methionyl aminopeptidase 1 (METAP1), mRNA. | NM_015143.1 |
| *MFNG* | Homo sapiens MFNG O-fucosylpeptide 3-beta-N-acetylglucosaminyltransferase (MFNG), mRNA. | NM_002405.2 |
| *MIF4GD* | Homo sapiens MIF4G domain containing (MIF4GD), mRNA. | NM_020679.2 |
| *MLLT10* | Homo sapiens myeloid/lymphoid or mixed-lineage leukemia (trithorax homolog, Drosophila); translocated to, 10 (MLLT10), transcript variant 1, mRNA. | NM_004641.2 |
| *MS4A7* | Homo sapiens membrane-spanning 4-domains, subfamily A, member 7 (MS4A7), transcript variant 3, mRNA. | NM_206939.1 |
| *MTMR12* | Homo sapiens myotubularin related protein 12 (MTMR12), mRNA. | NM_001040446.1 |
| *MXD4* | Homo sapiens MAX dimerization protein 4 (MXD4), mRNA. | NM_006454.2 |
| *NKTR* | Homo sapiens natural killer-tumor recognition sequence (NKTR), mRNA. | NM_005385.3 |
| *NUDT9* | Homo sapiens nudix (nucleoside diphosphate linked moiety X)-type motif 9 (NUDT9), transcript variant 3, mRNA. | NM_198038.1 |
| *NUP62* | Homo sapiens nucleoporin 62kDa (NUP62), transcript variant 2, mRNA. | NM_016553.3 |
| *NUP93* | Homo sapiens nucleoporin 93kDa (NUP93), mRNA. | NM_014669.2 |
| *OPN3* | Homo sapiens opsin 3 (encephalopsin, panopsin) (OPN3), mRNA. | NM_014322.2 |
| *ORAOV1* | Homo sapiens oral cancer overexpressed 1 (ORAOV1), mRNA. | NM_153451.2 |
| *PACSIN1* | Homo sapiens protein kinase C and casein kinase substrate in neurons 1 (PACSIN1), mRNA. | NM_020804.2 |
| *PAFAH1B1* | Homo sapiens platelet-activating factor acetylhydrolase, isoform Ib, alpha subunit 45kDa (PAFAH1B1), mRNA. | NM_000430.2 |
| *PARP1* | Homo sapiens poly (ADP-ribose) polymerase family, member 1 (PARP1), mRNA. | NM_001618.2 |
| *PAXIP1* | Homo sapiens PAX interacting (with transcription-activation domain) protein 1 (PAXIP1), mRNA. | NM_007349.3 |
| *PCBP1* | Homo sapiens poly(rC) binding protein 1 (PCBP1), mRNA. | NM_006196.2 |
| *PDHA1* | Homo sapiens pyruvate dehydrogenase (lipoamide) alpha 1 (PDHA1), mRNA. | NM_000284.1 |
| *PDHB* | Homo sapiens pyruvate dehydrogenase (lipoamide) beta (PDHB), mRNA. | NM_000925.1 |
| *PGRMC2* | Homo sapiens progesterone receptor membrane component 2 (PGRMC2), mRNA. | NM_006320.2 |
| *PHACTR4* | Homo sapiens phosphatase and actin regulator 4 (PHACTR4), transcript variant 1, mRNA. | NM_001048183.1 |
| *PHF17* | Homo sapiens PHD finger protein 17 (PHF17), transcript variant S, mRNA. | NM_024900.3 |
| *POFUT1* | Homo sapiens protein O-fucosyltransferase 1 (POFUT1), transcript variant 1, mRNA. | NM_015352.1 |
| *POLS* | Homo sapiens polymerase (DNA directed) sigma (POLS), mRNA. | NM_006999.3 |
| *PRKCH* | Homo sapiens protein kinase C, eta (PRKCH), mRNA. | NM_006255.3 |
| *PRNP* | Homo sapiens prion protein (p27-30) (Creutzfeldt-Jakob disease, Gerstmann-Strausler-Scheinker syndrome, fatal familial insomnia) (PRNP), transcript variant 3, mRNA. | NM_001080121.1 |
| *PRPSAP1* | Homo sapiens phosphoribosyl pyrophosphate synthetase-associated protein 1 (PRPSAP1), mRNA. | NM_002766.1 |
| *PTBP1* | Homo sapiens polypyrimidine tract binding protein 1 (PTBP1), transcript variant 1, mRNA. | NM_002819.3 |
| *PUS1* | Homo sapiens pseudouridylate synthase 1 (PUS1), transcript variant 2, mRNA. | NM_001002019.1 |
| *PYCR2* | Homo sapiens pyrroline-5-carboxylate reductase family, member 2 (PYCR2), mRNA. | NM_013328.2 |
| *QSOX2* | Homo sapiens quiescin Q6 sulfhydryl oxidase 2 (QSOX2), mRNA. | NM_181701.3 |
| *RAB11FIP3* | Homo sapiens RAB11 family interacting protein 3 (class II) (RAB11FIP3), mRNA. | NM_014700.2 |
| *RAB9A* | Homo sapiens RAB9A, member RAS oncogene family (RAB9A), mRNA. | NM_004251.3 |
| *RAD17* | Homo sapiens RAD17 homolog (S. pombe) (RAD17), transcript variant 3, mRNA. | NM_133340.1 |
| *RBL2* | Homo sapiens retinoblastoma-like 2 (p130) (RBL2), mRNA. | NM_005611.2 |
| *RCC2* | Homo sapiens regulator of chromosome condensation 2 (RCC2), mRNA. | NM_018715.1 |
| *RFTN1* | Homo sapiens raftlin, lipid raft linker 1 (RFTN1), mRNA. | NM_015150.1 |
| *RNF144* | Homo sapiens ring finger protein 144 (RNF144), mRNA. | NM_014746.2 |
| *RNF214* | Homo sapiens ring finger protein 214 (RNF214), transcript variant 1, mRNA. | NM_207343.2 |
| *RNF34* | Homo sapiens ring finger protein 34 (RNF34), transcript variant 2, mRNA. | NM_025126.2 |
| *RNPS1* | Homo sapiens RNA binding protein S1, serine-rich domain (RNPS1), transcript variant 2, mRNA. | NM_080594.1 |
| *RPA1* | Homo sapiens replication protein A1, 70kDa (RPA1), mRNA. | NM_002945.2 |
| *RPLP2* | Homo sapiens ribosomal protein, large, P2 (RPLP2), mRNA. | NM_001004.3 |
| *RTN1* | Homo sapiens reticulon 1 (RTN1), transcript variant 1, mRNA. | NM_021136.2 |
| *RUNX3* | Homo sapiens runt-related transcription factor 3 (RUNX3), transcript variant 2, mRNA. | NM_004350.2 |
| *SAMD3* | Homo sapiens sterile alpha motif domain containing 3 (SAMD3), transcript variant 1, mRNA. | NM_001017373.1 |
| *SBF1* | Homo sapiens SET binding factor 1 (SBF1), transcript variant 1, mRNA. | NM_002972.1 |
| *SEPT9* | Homo sapiens septin 9 (SEPT9), mRNA. | NM_006640.3 |
| *SFRS14* | Homo sapiens splicing factor, arginine/serine-rich 14 (SFRS14), transcript variant 2, mRNA. | NM_014884.2 |
| *SFRS2B* | Homo sapiens splicing factor, arginine/serine-rich 2B (SFRS2B), mRNA. | NM_032102.2 |
| *SFRS5* | Homo sapiens splicing factor, arginine/serine-rich 5 (SFRS5), transcript variant 2, mRNA. | NM_006925.3 |
| *SH2B3* | Homo sapiens SH2B adaptor protein 3 (SH2B3), mRNA. | NM_005475.1 |
| *SHMT1* | Homo sapiens serine hydroxymethyltransferase 1 (soluble) (SHMT1), transcript variant 1, mRNA. | NM_004169.3 |
| *SIAHBP1* | Homo sapiens fuse-binding protein-interacting repressor (SIAHBP1), transcript variant 2, mRNA. | NM_014281.3 |
| *SLBP* | Homo sapiens stem-loop (histone) binding protein (SLBP), mRNA. | NM_006527.2 |
| *SLC16A11* | Homo sapiens solute carrier family 16, member 11 (monocarboxylic acid transporter 11) (SLC16A11), mRNA. | NM_153357.1 |
| *SLC23A2* | Homo sapiens solute carrier family 23 (nucleobase transporters), member 2 (SLC23A2), transcript variant 2, mRNA. | NM_203327.1 |
| *SLC25A3* | Homo sapiens solute carrier family 25 (mitochondrial carrier; phosphate carrier), member 3 (SLC25A3), nuclear gene encoding mitochondrial protein, transcript variant 3, mRNA. | NM_213611.1 |
| *SLC25A5* | Homo sapiens solute carrier family 25 (mitochondrial carrier; adenine nucleotide translocator), member 5 (SLC25A5), mRNA. | NM_001152.1 |
| *SMAD7* | Homo sapiens SMAD family member 7 (SMAD7), mRNA. | NM_005904.2 |
| *SOX13* | Homo sapiens SRY (sex determining region Y)-box 13 (SOX13), mRNA. | NM_005686.2 |
| *SPG7* | Homo sapiens spastic paraplegia 7 (pure and complicated autosomal recessive) (SPG7), nuclear gene encoding mitochondrial protein, transcript variant 1, mRNA. | NM_003119.2 |
| *SPOCK2* | Homo sapiens sparc/osteonectin, cwcv and kazal-like domains proteoglycan (testican) 2 (SPOCK2), mRNA. | NM_014767.1 |
| *SPRYD5* | Homo sapiens SPRY domain containing 5 (SPRYD5), mRNA. | NM_032681.1 |
| *SRF* | Homo sapiens serum response factor (c-fos serum response element-binding transcription factor) (SRF), mRNA. | NM_003131.2 |
| *SRRM1* | Homo sapiens serine/arginine repetitive matrix 1 (SRRM1), mRNA. | NM_005839.3 |
| *SRRM1L* | PREDICTED: Homo sapiens serine/arginine repetitive matrix 1-like (SRRM1L), mRNA. | XM_932812.1 |
| *STK38* | Homo sapiens serine/threonine kinase 38 (STK38), mRNA. | NM_007271.2 |
| *STX2* | Homo sapiens syntaxin 2 (STX2), transcript variant 2, mRNA. | NM_194356.1 |
| *SUMO3* | Homo sapiens SMT3 suppressor of mif two 3 homolog 3 (S. cerevisiae) (SUMO3), mRNA. | NM_006936.2 |
| *SYPL1* | Homo sapiens synaptophysin-like 1 (SYPL1), transcript variant 1, mRNA. | NM_006754.2 |
| *TAF1L* | Homo sapiens TAF1 RNA polymerase II, TATA box binding protein (TBP)-associated factor, 210kDa-like (TAF1L), mRNA. | NM_153809.2 |
| *TEX261* | Homo sapiens testis expressed 261 (TEX261), mRNA. | NM_144582.2 |
| *TFG* | Homo sapiens TRK-fused gene (TFG), transcript variant 2, mRNA. | NM_001007565.1 |
| *TH1L* | Homo sapiens TH1-like (Drosophila) (TH1L), transcript variant 2, mRNA. | NM_016397.2 |
| *THAP11* | Homo sapiens THAP domain containing 11 (THAP11), mRNA. | NM_020457.2 |
| *THRAP4* | Homo sapiens thyroid hormone receptor associated protein 4 (THRAP4), transcript variant 1, mRNA. | NM_014815.3 |
| *TJAP1* | Homo sapiens tight junction associated protein 1 (peripheral) (TJAP1), mRNA. | NM_080604.1 |
| *TMEM109* | Homo sapiens transmembrane protein 109 (TMEM109), mRNA. | NM_024092.1 |
| *TMEM43* | Homo sapiens transmembrane protein 43 (TMEM43), mRNA. | NM_024334.1 |
| *TMEM5* | Homo sapiens transmembrane protein 5 (TMEM5), mRNA. | NM_014254.1 |
| *TMEM87A* | Homo sapiens transmembrane protein 87A (TMEM87A), mRNA. | NM_015497.2 |
| *TNFSF5IP1* | Homo sapiens tumor necrosis factor superfamily, member 5-induced protein 1 (TNFSF5IP1), mRNA. | NM_020232.3 |
| *TNPO1* | Homo sapiens transportin 1 (TNPO1), transcript variant 2, mRNA. | NM_153188.2 |
| *TRIAD3* | Homo sapiens TRIAD3 protein (TRIAD3), transcript variant 1, mRNA. | NM_207111.2 |
| *TRRAP* | Homo sapiens transformation/transcription domain-associated protein (TRRAP), mRNA. | NM_003496.1 |
| *TSHZ1* | Homo sapiens teashirt zinc finger homeobox 1 (TSHZ1), mRNA. | NM_005786.4 |
| *TSPYL1* | Homo sapiens TSPY-like 1 (TSPYL1), mRNA. | NM_003309.2 |
| *TTF2* | Homo sapiens transcription termination factor, RNA polymerase II (TTF2), mRNA. | NM_003594.3 |
| *U2AF2* | Homo sapiens U2 small nuclear RNA auxiliary factor 2 (U2AF2), transcript variant 1, mRNA. | NM_007279.2 |
| *UBAC2* | Homo sapiens UBA domain containing 2 (UBAC2), mRNA. | NM_177967.2 |
| *UBE2Q1* | Homo sapiens ubiquitin-conjugating enzyme E2Q (putative) 1 (UBE2Q1), mRNA. | NM_017582.5 |
| *UBXD8* | Homo sapiens UBX domain containing 8 (UBXD8), mRNA. | NM_014613.2 |
| *UVRAG* | Homo sapiens UV radiation resistance associated gene (UVRAG), mRNA. | NM_003369.3 |
| *WDR33* | Homo sapiens WD repeat domain 33 (WDR33), transcript variant 3, mRNA. | NM_001006623.1 |
| *WDR37* | Homo sapiens WD repeat domain 37 (WDR37), mRNA. | NM_014023.3 |
| *WDR4* | Homo sapiens WD repeat domain 4 (WDR4), transcript variant 2, mRNA. | NM_033661.3 |
| *WDR59* | Homo sapiens WD repeat domain 59 (WDR59), mRNA. | NM_030581.3 |
| *WDR67* | Homo sapiens WD repeat domain 67 (WDR67), mRNA. | NM_145647.2 |
| *WRNIP1* | Homo sapiens Werner helicase interacting protein 1 (WRNIP1), transcript variant 1, mRNA. | NM_020135.2 |
| *XPNPEP1* | Homo sapiens X-prolyl aminopeptidase (aminopeptidase P) 1, soluble (XPNPEP1), mRNA. | NM_020383.2 |
| *YEATS2* | Homo sapiens YEATS domain containing 2 (YEATS2), mRNA. | NM_018023.3 |
| *ZC3H7A* | Homo sapiens zinc finger CCCH-type containing 7A (ZC3H7A), mRNA. | NM_014153.2 |
| *ZCCHC14* | Homo sapiens zinc finger, CCHC domain containing 14 (ZCCHC14), mRNA. | NM_015144.2 |
| *ZFAND5* | Homo sapiens zinc finger, AN1-type domain 5 (ZFAND5), mRNA. | NM_006007.1 |
| *ZFP36* | Homo sapiens zinc finger protein 36, C3H type, homolog (mouse) (ZFP36), mRNA. | NM_003407.2 |
| *ZMYND8* | Homo sapiens zinc finger, MYND-type containing 8 (ZMYND8), transcript variant 1, mRNA. | NM_183047.1 |
| *ZNF207* | Homo sapiens zinc finger protein 207 (ZNF207), transcript variant 2, mRNA. | NM_001032293.2 |
| *ZNF212* | Homo sapiens zinc finger protein 212 (ZNF212), mRNA. | NM_012256.2 |
| *ZNF330* | Homo sapiens zinc finger protein 330 (ZNF330), mRNA. | NM_014487.3 |
| *ZNF664* | Homo sapiens zinc finger protein 664 (ZNF664), mRNA. | NM_152437.1 |
| *ZNF696* | Homo sapiens zinc finger protein 696 (ZNF696), mRNA. | NM_030895.1 |
