# Supplementary material for: Transcriptome Analysis Describing New Immunity and Defense Genes in Peripheral Blood Mononuclear Cells of Rheumatoid Arthritis Patients
Source: PLoS One. 2009 Aug 27;4(8):e6803. doi: 10.1371/journal.pone.0006803 (PMC2729373; doi:10.1371/journal.pone.0006803)
Supplement: Table S3 — List of 101 upregulated genes differentially expressed between RA patients and controls (0.14 MB DOC) [file pone.0006803.s003.doc]

**Table S3** List of 101 upregulated genes differentially expressed between RA patients and controls.

| **Gene**  **Symbol** | **Definition** | **Transcript Identifier** |
| --- | --- | --- |
| *ABCA1* | Homo sapiens ATP-binding cassette, sub-family A (ABC1), member 1 (ABCA1), mRNA. | NM_005502.2 |
| *ABCA7* | Homo sapiens ATP-binding cassette, sub-family A (ABC1), member 7 (ABCA7), mRNA. | NM_019112.3 |
| *AIF1* | Homo sapiens allograft inflammatory factor 1 (AIF1), transcript variant 3, mRNA. | NM_001623.3 |
| *ANKRD22* | Homo sapiens ankyrin repeat domain 22 (ANKRD22), mRNA. | NM_144590.2 |
| *ARG1* | Homo sapiens arginase, liver (ARG1), mRNA. | NM_000045.2 |
| *ATG10* | Homo sapiens ATG10 autophagy related 10 homolog (S. cerevisiae) (ATG10), mRNA. | NM_031482.3 |
| *ATP5J* | Homo sapiens ATP synthase, H+ transporting, mitochondrial F0 complex, subunit F6 (ATP5J), nuclear gene encoding mitochondrial protein, transcript variant 1, mRNA. | NM_001003703.1 |
| *ATP6V0E1* | Homo sapiens ATPase, H+ transporting, lysosomal 9kDa, V0 subunit e1 (ATP6V0E1), mRNA. | NM_003945.3 |
| *ATP6V1D* | Homo sapiens ATPase, H+ transporting, lysosomal 34kDa, V1 subunit D (ATP6V1D), mRNA. | NM_015994.2 |
| *BAG4* | Homo sapiens BCL2-associated athanogene 4 (BAG4), mRNA. | NM_004874.2 |
| *BLOC1S1* | Homo sapiens biogenesis of lysosome-related organelles complex-1, subunit 1 (BLOC1S1), mRNA. | NM_001487.1 |
| *BUD31* | Homo sapiens BUD31 homolog (S. cerevisiae) (BUD31), mRNA. | NM_003910.2 |
| *C14orf2* | Homo sapiens chromosome 14 open reading frame 2 (C14orf2), mRNA. | NM_004894.1 |
| *C16orf7* | Homo sapiens chromosome 16 open reading frame 7 (C16orf7), mRNA. | NM_004913.2 |
| *C19orf59* | Homo sapiens chromosome 19 open reading frame 59 (C19orf59), mRNA. | NM_174918.2 |
| *C5orf32* | Homo sapiens chromosome 5 open reading frame 32 (C5orf32), mRNA. | NM_032412.3 |
| *CAMP* | Homo sapiens cathelicidin antimicrobial peptide (CAMP), mRNA. | NM_004345.3 |
| *CBS* | Homo sapiens cystathionine-beta-synthase (CBS), mRNA. | NM_000071.1 |
| *CBX3* | Homo sapiens chromobox homolog 3 (HP1 gamma homolog, Drosophila) (CBX3), transcript variant 2, mRNA. | NM_016587.2 |
| *CCDC72* | Homo sapiens coiled-coil domain containing 72 (CCDC72), mRNA. | NM_015933.3 |
| *CITED4* | Homo sapiens Cbp/p300-interacting transactivator, with Glu/Asp-rich carboxy-terminal domain, 4 (CITED4), mRNA. | NM_133467.2 |
| *CKLF* | Homo sapiens chemokine-like factor (CKLF), transcript variant 5, mRNA. | NM_001040138.1 |
| *CNOT1* | Homo sapiens CCR4-NOT transcription complex, subunit 1 (CNOT1), transcript variant 2, mRNA. | NM_206999.1 |
| *COX6A1* | Homo sapiens cytochrome c oxidase subunit VIa polypeptide 1 (COX6A1), nuclear gene encoding mitochondrial protein, mRNA. | NM_004373.2 |
| *COX7A2* | Homo sapiens cytochrome c oxidase subunit VIIa polypeptide 2 (liver) (COX7A2), mRNA. | NM_001865.2 |
| *COX7C* | Homo sapiens cytochrome c oxidase subunit VIIc (COX7C), nuclear gene encoding mitochondrial protein, mRNA. | NM_001867.2 |
| *CPEB3* | Homo sapiens cytoplasmic polyadenylation element binding protein 3 (CPEB3), mRNA. | NM_014912.3 |
| *DDEF1* | Homo sapiens development and differentiation enhancing factor 1 (DDEF1), mRNA. | NM_018482.2 |
| *DDIT3* | Homo sapiens DNA-damage-inducible transcript 3 (DDIT3), mRNA. | NM_004083.4 |
| *DKFZp761E198* | Homo sapiens DKFZp761E198 protein (DKFZp761E198), mRNA. | NM_138368.3 |
| *ERH* | Homo sapiens enhancer of rudimentary homolog (Drosophila) (ERH), mRNA. | NM_004450.1 |
| *F5* | Homo sapiens coagulation factor V (proaccelerin, labile factor) (F5), mRNA. | NM_000130.4 |
| *FBN2* | Homo sapiens fibrillin 2 (congenital contractural arachnodactyly) (FBN2), mRNA. | NM_001999.3 |
| *GAPDH* | Homo sapiens glyceraldehyde-3-phosphate dehydrogenase (GAPDH), mRNA. | NM_002046.3 |
| *GDPD3* | Homo sapiens glycerophosphodiester phosphodiesterase domain containing 3 (GDPD3), mRNA. | NM_024307.2 |
| *GLRX* | Homo sapiens glutaredoxin (thioltransferase) (GLRX), mRNA. | NM_002064.1 |
| *GMFG* | Homo sapiens glia maturation factor, gamma (GMFG), mRNA. | NM_004877.1 |
| *GTF2B* | Homo sapiens general transcription factor IIB (GTF2B), mRNA. | NM_001514.3 |
| *HK3* | Homo sapiens hexokinase 3 (white cell) (HK3), nuclear gene encoding mitochondrial protein, mRNA. | NM_002115.1 |
| *HNMT* | Homo sapiens histamine N-methyltransferase (HNMT), transcript variant 2, mRNA. | NM_001024074.1 |
| *ING4* | Homo sapiens inhibitor of growth family, member 4 (ING4), mRNA. | NM_016162.2 |
| *JARID1B* | Homo sapiens jumonji, AT rich interactive domain 1B (JARID1B), mRNA. | NM_006618.3 |
| *KCTD20* | Homo sapiens potassium channel tetramerisation domain containing 20 (KCTD20), mRNA. | NM_173562.3 |
| *KIAA1530* | Homo sapiens KIAA1530 protein (KIAA1530), mRNA. | NM_020894.1 |
| *LHFPL2* | Homo sapiens lipoma HMGIC fusion partner-like 2 (LHFPL2), mRNA. | NM_005779.2 |
| *LOC432369* | Homo sapiens ATP synthase, H+ transporting, mitochondrial F1 complex, epsilon subunit pseudogene 2 (LOC432369) on chromosome 13. | NR_002162.1 |
| *LRRFIP1* | Homo sapiens leucine rich repeat (in FLII) interacting protein 1 (LRRFIP1), mRNA. | NM_004735.2 |
| *LSM10* | Homo sapiens LSM10, U7 small nuclear RNA associated (LSM10), mRNA. | NM_032881.1 |
| *LSM3* | Homo sapiens LSM3 homolog, U6 small nuclear RNA associated (S. cerevisiae) (LSM3), mRNA. | NM_014463.1 |
| *LY96* | Homo sapiens lymphocyte antigen 96 (LY96), mRNA. | NM_015364.2 |
| *MRPL33* | Homo sapiens mitochondrial ribosomal protein L33 (MRPL33), nuclear gene encoding mitochondrial protein, transcript variant 1, mRNA. | NM_004891.3 |
| *MRPS18C* | Homo sapiens mitochondrial ribosomal protein S18C (MRPS18C), nuclear gene encoding mitochondrial protein, mRNA. | NM_016067.1 |
| *MSL3L1* | Homo sapiens male-specific lethal 3-like 1 (Drosophila) (MSL3L1), transcript variant 1, mRNA. | NM_078629.1 |
| *MXD3* | Homo sapiens MAX dimerization protein 3 (MXD3), mRNA. | NM_031300.2 |
| *MYL6* | Homo sapiens myosin, light chain 6, alkali, smooth muscle and non-muscle (MYL6), transcript variant 1, mRNA. | NM_021019.3 |
| *MYL6B* | Homo sapiens myosin, light chain 6B, alkali, smooth muscle and non-muscle (MYL6B), mRNA. | NM_002475.3 |
| *NBEAL2* | Homo sapiens neurobeachin-like 2 (NBEAL2), mRNA. | NM_015175.1 |
| *NCF4* | Homo sapiens neutrophil cytosolic factor 4, 40kDa (NCF4), transcript variant 2, mRNA. | NM_013416.2 |
| *NDUFA1* | Homo sapiens NADH dehydrogenase (ubiquinone) 1 alpha subcomplex, 1, 7.5kDa (NDUFA1), nuclear gene encoding mitochondrial protein, mRNA. | NM_004541.2 |
| *NDUFB3* | Homo sapiens NADH dehydrogenase (ubiquinone) 1 beta subcomplex, 3, 12kDa (NDUFB3), mRNA. | NM_002491.1 |
| *NFAT5* | Homo sapiens nuclear factor of activated T-cells 5, tonicity-responsive (NFAT5), transcript variant 5, mRNA. | NM_173215.1 |
| *NRD1* | Homo sapiens nardilysin (N-arginine dibasic convertase) (NRD1), mRNA. | NM_002525.1 |
| *NUFIP2* | Homo sapiens nuclear fragile X mental retardation protein interacting protein 2 (NUFIP2), mRNA. | NM_020772.1 |
| *NUP214* | Homo sapiens nucleoporin 214kDa (NUP214), mRNA. | NM_005085.2 |
| *ORM1* | Homo sapiens orosomucoid 1 (ORM1), mRNA. | NM_000607.1 |
| *ORM2* | Homo sapiens orosomucoid 2 (ORM2), mRNA. | NM_000608.2 |
| *PGLYRP1* | Homo sapiens peptidoglycan recognition protein 1 (PGLYRP1), mRNA. | NM_005091.1 |
| *PHF20L1* | Homo sapiens PHD finger protein 20-like 1 (PHF20L1), transcript variant 2, mRNA. | NM_032205.3 |
| *POLE4* | Homo sapiens polymerase (DNA-directed), epsilon 4 (p12 subunit) (POLE4), mRNA. | NM_019896.2 |
| *PPP2R3C* | Homo sapiens protein phosphatase 2 (formerly 2A), regulatory subunit B'', gamma (PPP2R3C), mRNA. | NM_017917.2 |
| *PTGES3* | Homo sapiens prostaglandin E synthase 3 (cytosolic) (PTGES3), mRNA. | NM_006601.4 |
| *PTRH2* | Homo sapiens peptidyl-tRNA hydrolase 2 (PTRH2), nuclear gene encoding mitochondrial protein, mRNA. | NM_016077.3 |
| *RAB24* | Homo sapiens RAB24, member RAS oncogene family (RAB24), transcript variant 2, mRNA. | NM_130781.1 |
| *RBP7* | Homo sapiens retinol binding protein 7, cellular (RBP7), mRNA. | NM_052960.1 |
| *RNASE2* | Homo sapiens ribonuclease, RNase A family, 2 (liver, eosinophil-derived neurotoxin) (RNASE2), mRNA. | NM_002934.2 |
| *RNASE3* | Homo sapiens ribonuclease, RNase A family, 3 (eosinophil cationic protein) (RNASE3), mRNA. | NM_002935.2 |
| *RRAGD* | Homo sapiens Ras-related GTP binding D (RRAGD), mRNA. | NM_021244.3 |
| *S100A12* | Homo sapiens S100 calcium binding protein A12 (S100A12), mRNA. | NM_005621.1 |
| *S100A8* | Homo sapiens S100 calcium binding protein A8 (S100A8), mRNA. | NM_002964.3 |
| *S100A9* | Homo sapiens S100 calcium binding protein A9 (S100A9), mRNA. | NM_002965.3 |
| *SAMD4B* | Homo sapiens sterile alpha motif domain containing 4B (SAMD4B), mRNA. | NM_018028.2 |
| *SCNM1* | Homo sapiens sodium channel modifier 1 (SCNM1), mRNA. | NM_024041.2 |
| *SF3B14* | Homo sapiens splicing factor 3B, 14 kDa subunit (SF3B14), mRNA. | NM_016047.3 |
| *SLC11A1* | Homo sapiens solute carrier family 11 (proton-coupled divalent metal ion transporters), member 1 (SLC11A1), mRNA. | NM_000578.3 |
| *SLC22A17* | Homo sapiens solute carrier family 22 (organic cation transporter), member 17 (SLC22A17), transcript variant 2, mRNA. | NM_016609.3 |
| *SLPI* | Homo sapiens secretory leukocyte peptidase inhibitor (SLPI), mRNA. | NM_003064.2 |
| *SRPK1* | Homo sapiens SFRS protein kinase 1 (SRPK1), mRNA. | NM_003137.3 |
| *SSH1* | Homo sapiens slingshot homolog 1 (Drosophila) (SSH1), mRNA. | NM_018984.2 |
| *STX10* | Homo sapiens syntaxin 10 (STX10), mRNA. | NM_003765.1 |
| *STX6* | Homo sapiens syntaxin 6 (STX6), mRNA. | NM_005819.4 |
| *SULT2B1* | Homo sapiens sulfotransferase family, cytosolic, 2B, member 1 (SULT2B1), transcript variant 2, mRNA. | NM_177973.1 |
| *TCEB2* | Homo sapiens transcription elongation factor B (SIII), polypeptide 2 (18kDa, elongin B) (TCEB2), transcript variant 1, mRNA. | NM_007108.2 |
| *THEM2* | Homo sapiens thioesterase superfamily member 2 (THEM2), mRNA. | NM_018473.2 |
| *TLR5* | Homo sapiens toll-like receptor 5 (TLR5), mRNA. | NM_003268.4 |
| *TMCC3* | Homo sapiens transmembrane and coiled-coil domain family 3 (TMCC3), mRNA. | NM_020698.1 |
| *TMEM97* | Homo sapiens transmembrane protein 97 (TMEM97), mRNA. | NM_014573.2 |
| *TNPO1* | Homo sapiens transportin 1 (TNPO1), transcript variant 2, mRNA. | NM_153188.2 |
| *TXN* | Homo sapiens thioredoxin (TXN), mRNA. | NM_003329.2 |
| *UBL5* | Homo sapiens ubiquitin-like 5 (UBL5), transcript variant 2, mRNA. | NM_001048241.1 |
| *VPS25* | Homo sapiens vacuolar protein sorting 25 homolog (S. cerevisiae) (VPS25), mRNA. | NM_032353.2 |
| *ZNF148* | Homo sapiens zinc finger protein 148 (ZNF148), mRNA. | NM_021964.2 |
